# Tuberculosis Infection Mimicking Brain Metastatic Malignancy Lesions in an Elderly Male

**DOI:** 10.1155/2013/146032

**Published:** 2013-07-24

**Authors:** Dimitrios Anyfantakis, Ageliki Damianaki, Maria Kokosi, Emmanouil K. Symvoulakis, Serafim Kastanakis

**Affiliations:** ^1^Primary Health Care Centre of Kissamos, 74100 Chania, Crete, Greece; ^2^Department of Internal Medicine, Saint George General Hospital of Chania, 73100 Chania, Crete, Greece; ^3^Private Family Practice Unit in Heraklion, 71303 Heraklion, Crete, Greece

## Abstract

An 83-year-old Caucasian Greek man was referred by his general practitioner to the emergency department of the general hospital in Crete because of seizures and agitation. His past medical history was negative for any neurological or medical condition. Electroencephalogram showed a bradyarrhythmic theta activity, without evidence of any focal or other specific abnormality. Magnetic resonance imaging of the brain demonstrated a number of diffuse nodular lesions and moderate perivascular edema. An axillary lymph node fine needle aspiration cytology suggested a granulomatous lymphadenitis along with signs of tuberculous infiltration. Tuberculin skin test was positive. We report a rare case of extrapulmonary tuberculosis mimicking brain metastatic lesions.

## 1. Introduction

Human tuberculosis (TB) represents a serious infectious disease with a significant global burden [1]. Remarkably, it has been reported that TB was responsible for 8.7 million new cases in 2011 with 1.4 million deaths worldwide [1]. Extrapulmonary manifestations of tuberculosis occur frequently among immunosuppressed patients [2]. TB of the central nervous system occurs rarely [3] and may present with different clinical and imaging patterns leading to diagnostic challenges [4]. Here we report an unusual presentation of extrapulmonary TB infection initially misdiagnosed as brain metastatic lesions.

## 2. Case Presentation

**Figure 1:**
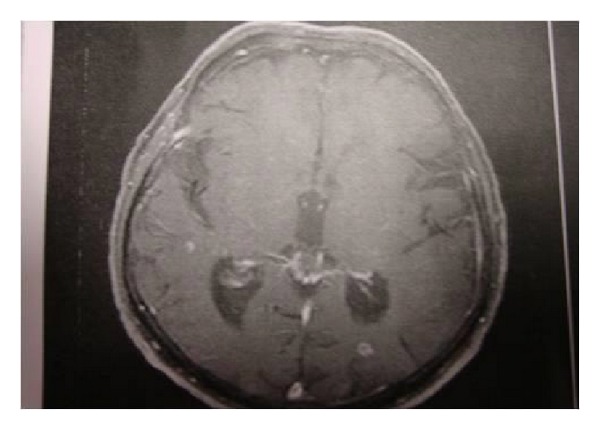
Axial MRI of the brain showing diffuse lesions mimicking secondary tumours.

An 83-year-old Caucasian Greek man was referred to the emergency department of the Saint George General Hospital of Chania, Crete, by his general practitioner because of seizures and agitation. His past medical history was negative for any neurological or medical condition. A four-week history of low-grade fever (up to 37.8 grade Celsius) has been reported. At the time of admission, he was alert and well oriented in place and time. He denied any history of smoking, drug or alcohol abuse. The blood pressure was 150/110 mmHg, the pulse was 88 beats per minute, and the respiratory rate was 15 breaths per minute. A complete blood cell count revealed the following: white blood cells, 7.140 cells/*μ*L; hematocrit, 35.1%; haemoglobin, 11.4 g/dL; and platelet counts, 381 cells/mL. Measurement of serum inflammatory markers showed elevated levels of erythrocyte sedimentation rate (41 mm/h) and of C-reactive protein (4.7 mg/dL). Renal and liver function tests were normal. Electrocardiogram revealed sinus rhythm without any pathological finding. Electroencephalogram showed a bradyarrhythmic theta activity, without evidence of any focal or other specific abnormality. Magnetic resonance imaging (MRI) of the brain demonstrated a number of diffuse nodular lesions located in the left occipital lobe, in the temporal lobes and in the right cerebellar hemisphere, and it demonstrated moderate peri-vascular edema (Figure 1). These findings were attributed to probable metastases, and the patient was referred to the oncology department. Tumor marker analysis revealed that alpha-fetoprotein (CA 15.3, CEA, CA 19.9) and the total PSA levels were all within normal limits. A lumbar punction was also performed. Cytological examination of cerebrospinal fluid (CSF) was negative for malignancy. A consecutive CSF analysis showed lymphocytic pleocytosis (lymphocytes, 94%; neutrophils, 4%) with moderately elevated protein levels (0.664 g/L) and low CSF glucose levels (2.27 mmol/L).

**Figure 2:**
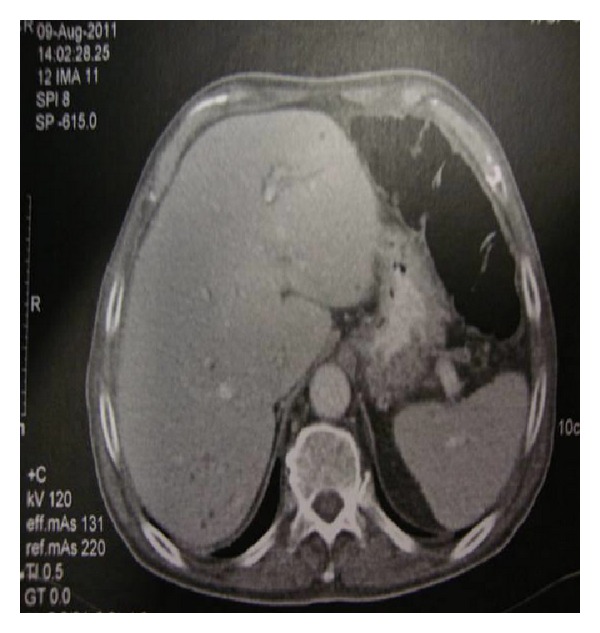
Abdominal CT showing hypodense lesions located in the liver and spleen and enlarged retroperitoneal lymph nodes.

Abdominal CT scan disclosed retroperitoneal lymphadenopathy and diffuse hypodense lesions located in hepatic and splenic parenchyma (Figure 2). Further imaging with CT thoracic scan showed an abnormal enlargement of the right axillary lymph nodes. Consequently a fine needle aspiration of the right axillary lymph node was performed. Cytological examination showed no evidence of malignancy. Findings were suggestive of a granulomatous lymphadenitis with signs of tuberculous inflammatory infiltration. A tuberculin skin test was performed and was found positive (20 mm).

**Figure 3:**
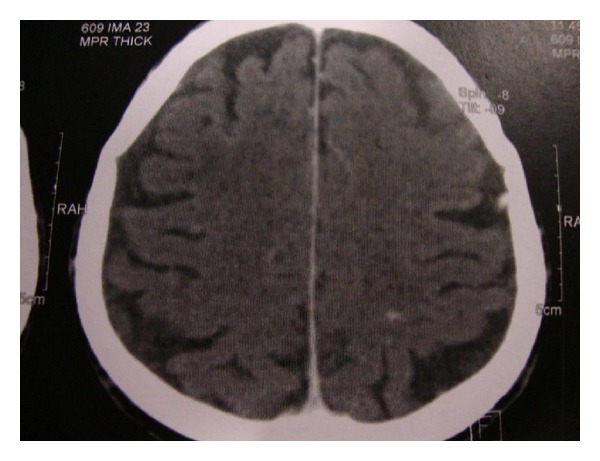
Axial MRI of the brain following 3 months of anti-TB therapy demonstrating an important resolution of the previous brain lesions.

**Figure 4:**
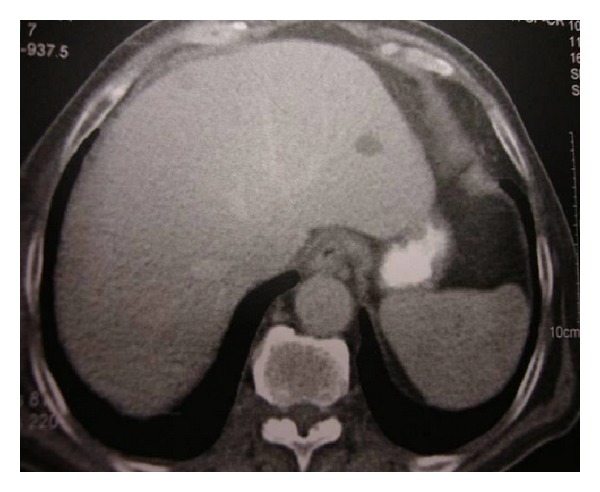
Abdominal CT imaging 3 months after administration of anti-TB agents, showing reduction of the hypodense lesions and of the retroperitoneal lymphadenopathy.

Anti-TB treatment with the standard drug regimen was initiated (isoniazid 300 mg daily, rifampicin 600 mg daily, pyrazinamide 2 g daily, and ethambutol 1 g daily). After 3 months of anti-TB therapy, brain MRI (Figure 3) and abdominal CT imaging (Figure 4) showed a significant resolution of brain lesions and reduction of the retroperitoneal lymph nodes swelling, respectively.

## 3. Discussion

TB infection of the central nervous system (CNS) is a severe, potentially fatal form of the disease that usually affects young children and immunosuppressed individuals [3]. Other predisposing factors include malnutrition, alcohol abuse, and malignancies [4]. It accounts for approximately 1% of all cases of TB [3]. Historically, Rich and McCordock first reported data regarding pathogenesis of tuberculous meningitis in 1933 [5].

Tuberculous meningitis is a pathological manifestation occurring more frequently, followed by tuberculoma, tuberculous abscess, cerebral miliary tuberculosis, tuberculous encephalitis, and tuberculous arteritis [4]. Small tuberculous foci, which are also named Rich foci, are developed in the meninges, brain, or the spinal cord [3]. The increase of tubercles in the parenchyma of the brain without a rupture into the subarachnoid space leads to the development of tuberculomas [3]. They often appear as solitary lesions, although multiple tuberculomas may also develop [3]. The formation of brain abscess represents an unusual manifestation of CNS TB [3]. These can present as single or multiple lesions [6]. Since clinical symptoms are nonspecific, prompt diagnosis is challenging [4]. Malaise, mood disturbance, and headache may be the initial manifestations of central nervous system TB [4], followed by the classic symptoms of meningitis with vomiting, neck stiffness, and focal neurological deficits [7]. A previous history of exposure to TB or a positive tuberculin skin test may raise the diagnostic suspicion for TB CNS infection [7].

Tuberculomas and tuberculous brain abscesses follow a chronic clinical course from weeks to months and may be manifested with seizures, headaches, papilledema, or other signs of increased intracranial pressure [3]. Tuberculous brain abscesses are developed more acute (from 1 week to 3 months) than tuberculomas, and they are associated with fever, headaches, and focal neurological deficits [8]. In regards to the most suitable radiological approach for the detection of meningeal and parenchymal lesions in the context of CNS TB, contrast-enhanced MRI is considered superior to CT [9].

An initial therapeutic assessment of CNS TB consists of a two-month administration of the following pharmacological regimen: isoniazid, rifampicin, pyrazinamide, and ethambutol [3, 10]. A maintenance therapy of seven to ten months with isoniazid and rifampicin is suggested after that [3]. Parenteral forms of isoniazid, rifampicin, aminoglycosides and fluoroquinolones are available for intravenous administration in case that the patient presents an altered mental status [3].

We presented an unusual case of CNS TB in a patient without any apparent predisposing risk factors such as previous exposure to TB, malignancy, malnutrition, or alcohol abuse. Our case also highlights the necessity for the physicians to maintain a high level of suspicion for the unusual presentations of extrapulmonary TB especially in countries where the disease is endemic [11]. Much of the diagnostic management of this case was orientated to detect the primary malignancy site of an apparent brain metastatic disease, presumably leading to medical staff and patient uncertainty. New cases of TB infection are potentially present since migration or economic vulnerability can enhance this eventuality. In Greece, the crisis is expected to challenge surveillance mechanisms, and an integrated plan to prevent underreporting, and its consequences, is needed [12]. This case report offers an educational message for the eventuality of a serious communicable disease diagnosis which for many physicians is out of their practicing awareness. The diagnostic delays may lead to patient and community health safety.
